# Sociodemographic, Reproductive, and Clinical Profile of Patients With an Unhealthy Cervix: An Observational Study

**DOI:** 10.7759/cureus.100298

**Published:** 2025-12-28

**Authors:** Shubhalaxmi Nene, Indrani Dutta, Moumita Kundu

**Affiliations:** 1 Obstetrics and Gynaecology, Abhishek I Mishra Memorial Medical College and Research, Bhilai, IND; 2 Obstetrics and Gynaecology, Manipal Tata Medical College, Jamshedpur, IND; 3 Community Medicine, KPC Medical College and Hospital, Kolkata, IND

**Keywords:** papanicolaou test, premalignant lesions, reproductive health, screening programs, socioeconomic factors, vaginal discharge

## Abstract

Background

Cervical cancer is a major public health challenge in India. The term "unhealthy cervix" encompasses a spectrum of cervical abnormalities detectable through visual examination, ranging from inflammatory conditions to premalignant and malignant lesions. This study was conducted with the aim of describing the sociodemographic, reproductive, and clinical characteristics of patients presenting with an unhealthy cervix at tertiary care centers in Central and Eastern India.

Methods

This hospital-based observational study was conducted from March 2025 to August 2025 at two tertiary care hospitals. A total of 280 women aged 21-65 years presenting with complaints suggestive of an unhealthy cervix were enrolled using consecutive sampling. Data on sociodemographic characteristics, reproductive history, and clinical presentation were collected using a predesigned proforma. All participants underwent a comprehensive pelvic examination and Papanicolaou (Pap) test.

Results

Among 280 patients, the mean age was 38.6 ± 9.2 years, with 163 (58.2%) from rural areas and 146 (52.1%) illiterate. The majority (n = 193, 68.9%) belonged to lower socioeconomic classes. Multiparity was reported by 215 (76.8%) participants. White vaginal discharge was the most common presenting complaint (n = 152, 54.3%). A Pap test revealed benign inflammatory changes in 175 (62.5%) and abnormal cytology in 26 (9.3%) participants. Cervical erosion was the most frequent diagnosis (n = 75, 26.8%), while premalignant conditions were identified in 26 (9.3%) participants. Abnormal Pap test results were significantly associated with illiteracy (p = 0.015), lower socioeconomic status (p = 0.042), teenage pregnancy (p = 0.018), multiple sexual partners (p = 0.003), history of sexually transmitted infections (p < 0.001), and long-term oral contraceptive use (p = 0.006).

Conclusion

This study demonstrates a high burden of cervical pathology among symptomatic women, particularly those from lower socioeconomic backgrounds. The findings emphasize the need for comprehensive cervical cancer prevention strategies targeting high-risk populations through enhanced screening programs, health education initiatives, and strengthening of healthcare infrastructure in underserved areas.

## Introduction

Cervical cancer is a major public health concern in India, being the second most common cancer among women and a leading cause of cancer-related mortality [1]. Despite being largely preventable through early detection and screening, the burden disproportionately affects low- and middle-income countries, where approximately 94% of cervical cancer deaths occur annually [2]. India accounts for approximately one-quarter of the global cervical cancer burden, with cervical cancer constituting 18.3% of new cases of cancer and 9.4% of all malignancies among women in 2020 [1,3].

The term "unhealthy cervix" refers to a clinical assessment made through visual examination, where the cervix exhibits abnormal features such as unusual growth patterns, ulceration, or aberrant vascular appearance [4]. Recognition of an unhealthy cervix through routine examination serves as a critical entry point for further diagnostic evaluation and timely intervention [3]. The prevalence is particularly pronounced among rural women of low socioeconomic status and those residing in urban slums, who face multiple risk factors including early marriage, multiparity, poor nutritional status, limited contraceptive awareness, and inadequate access to screening programs [4-6].

The Papanicolaou test, also known as the Pap test, has been the cornerstone of cervical cancer screening for decades, offering high specificity and being cost-effective, non-invasive, and easily implementable [7,8]. However, its sensitivity for detecting precancerous lesions remains suboptimal, particularly in resource-limited settings [9]. Understanding the sociodemographic and clinical profile of women presenting with an unhealthy cervix is essential for planning targeted screening and intervention strategies for cervical cancer prevention and control. Therefore, this study was conducted to describe the sociodemographic, reproductive, and clinical profile of patients presenting with an unhealthy cervix at tertiary care centers.

## Materials and methods

We undertook an observational study over six months from March 2025 to August 2025, which was carried out simultaneously at two tertiary care hospitals: one in Bhilai, Chhattisgarh (Central India), and another in Jamshedpur, Jharkhand (Eastern India), specifically in the Department of Obstetrics and Gynaecology at Abhishek I Mishra Memorial Medical College and Research and Manipal Tata Medical College. The study included women aged 21-65 years presenting to the Gynaecology and Obstetrics outpatient departments with complaints suggestive of an unhealthy cervix, such as vaginal discharge, menstrual disorders, post-coital bleeding, post-menopausal bleeding, and intermenstrual bleeding. Women not providing informed written consent, those with active severe vaginal bleeding at the time of examination, pregnant women, women with frank invasive cervical cancer on clinical examination, and those with a previous history of cervical malignancy or treatment for cervical lesions were excluded from the study.

During the study period, a consecutive sampling strategy was used to enroll 280 patients from both institutions who met the eligibility criteria. Data were collected using a predesigned, pretested proforma encompassing demographic and clinical data, clinical examination procedures, and screening diagnostic procedures. All participants underwent a comprehensive per speculum examination to assess cervical and vaginal conditions. Presenting complaints, including the nature of vaginal discharge, abnormal bleeding patterns, and other gynecological symptoms, were systematically recorded. Following informed consent and clinical examination, a Pap smear was obtained by a trained gynecologist from all participants, following which the cervical samples were sent to the Department of Pathology. The slides were evaluated by two independent pathologists, and in cases of discordant results, a consensus was reached through joint discussion. Results were reported according to the Bethesda System [10]. Positive Pap test results were defined as atypical squamous cells of undetermined significance (ASCUS), low-grade squamous intraepithelial lesion (LSIL), high-grade squamous intraepithelial lesion (HSIL), or invasive cancer. The final diagnosis was based on clinical findings, Pap test results, and additional investigations as warranted.

Microsoft Excel 2016 (Microsoft Corp., Redmond, WA, US) was used to organize the data, while SPSS version 26.0 (IBM Corp., Armonk, NY, US) was used for analysis. Categorical variables were shown as frequencies and percentages, while continuous variables were given as mean ± standard deviation. For continuous variables, the independent sample t-test and Mann-Whitney U test were utilized; for categorical variables, the Chi-squared test and Fisher's exact test were utilized. The threshold for statistical significance was established at p < 0.05.

The study protocol was approved by the Institutional Ethics Committees of both participating hospitals prior to initiation of data collection. All participants were provided detailed information about the study purpose, and written informed consent was obtained in local languages.

## Results

Final analysis was conducted on 280 patients who met the eligibility criteria and completed all study procedures. Table 1 presents the sociodemographic and reproductive characteristics of the study participants. The mean age of participants was 38.6 ± 9.2 years, with the majority (n = 119, 42.5%) aged between 31 and 40 years. More than half of the participants (n = 163, 58.2%) were from rural areas. A substantial proportion were illiterate (n = 146, 52.1%), and 197 participants (68.9%) belonged to lower socioeconomic classes (Classes IV and V).

**Table 1:** Sociodemographic and reproductive characteristics of study participants (N = 280)

Characteristics	Categories	Frequency (n)	Percentage (%)
Age (years)	≤30	68	24.3
	31-40	119	42.5
	41-50	64	22.9
	51-60	23	8.2
	>60	6	2.1
Residence	Rural	163	58.2
	Urban	117	41.8
Literacy	Literate	134	47.9
	Illiterate	146	52.1
Socioeconomic status	Class I (upper class)	12	4.3
	Class II (upper middle class)	23	8.2
	Class III (middle class)	52	18.6
	Class IV (lower middle class)	118	42.1
	Class V (lower class)	75	26.8
Parity	Nulliparous	18	6.4
	Primiparous	47	16.8
	Multiparous	215	76.8
Teenage pregnancy	Yes	35	12.5
Multiple sexual partners	Yes	10	3.6
Past history of sexually transmitted infections	Yes	25	8.9
Long-term use of oral contraceptive pills (≥5 years)	Yes	51	18.2

Regarding reproductive characteristics, the majority of participants were multiparous (n = 215, 76.8%). Teenage pregnancy was reported by 35 participants (12.5%). A history of multiple sexual partners was reported by 10 participants (3.6%), while 25 reported a past history of sexually transmitted infections (STIs) (8.9%). Long-term oral contraceptive pill (OCP) use (≥5 years) was reported by 51 participants (18.2%) (Table 1).

Table 2 summarizes the presenting complaints and Pap test findings among study participants. White vaginal discharge was the most common presenting complaint (n = 152, 54.3%). Blood-tinged discharge was reported by 38 participants (13.6%). Pap test examination revealed that the majority (n = 175, 62.5%) had benign inflammatory changes. Normal cytology was found in 79 participants (28.2%). Abnormal Pap test results included ASCUS (n = 10, 3.6%), LSIL (n = 9, 3.2%), and HSIL (n = 7, 2.5%).

**Table 2:** Presenting complaints and Pap test findings among study participants (N = 280) *Multiple complaints may be present in a single participant ASCUS: atypical squamous cells of undetermined significance; LSIL: low-grade squamous intraepithelial lesion; HSIL: high-grade squamous intraepithelial lesion

Characteristics	Categories	Frequency (n)	Percentage (%)
Presenting complaints*	White vaginal discharge	152	54.3
	Foul-smelling discharge	53	18.9
	Blood-stained discharge	38	13.6
	Intermenstrual bleeding	80	28.6
	Post-coital bleeding	43	15.4
	Post-menopausal bleeding	35	12.5
	Pelvic pain	62	22.1
Pap test findings	Normal	79	28.2
	Benign inflammation	175	62.5
	ASCUS	10	3.6
	LSIL	9	3.2
	HSIL	7	2.5

The most frequently occurring conditions were cervical erosion (26.8%) and bacterial vaginosis (15.7%). Candidal vaginitis was diagnosed in 39 participants (13.9%). Premalignant conditions (ASCUS/LSIL/HSIL) were identified in 26 participants (9.3%) (Table 3).

**Table 3:** Final diagnosis based on clinical and Pap test results (N = 280) ASCUS: atypical squamous cells of undetermined significance; LSIL: low-grade squamous intraepithelial lesion; HSIL: high-grade squamous intraepithelial lesion

Diagnosis	Frequency (n)	Percentage (%)
Physiological leukorrhea	49	17.5
Non-specific vaginitis	24	8.6
Bacterial vaginosis	44	15.7
Candidal vaginitis	39	13.9
Cervical erosion	75	26.8
Chronic pelvic inflammatory disease	18	6.4
Cervical polyp	5	1.8
Premalignant conditions (ASCUS/LSIL/HSIL)	26	9.3

Table 4 identifies factors associated with cervical premalignant and malignant conditions (abnormal Pap test results). Compared to those with normal or benign inflammatory Pap test results, a significantly higher proportion of patients with abnormal Pap test results belonged to lower socioeconomic classes (84.6% vs. 67.3%; p = 0.042), were illiterate (73.1% vs. 49.8%; p = 0.015), had teenage pregnancy (26.9% vs. 11.1%; p = 0.018), reported having multiple sexual partners (15.4% vs. 2.5%; p = 0.003), had past history of STI (30.8% vs. 7.1%; p < 0.001), and reported long-term use of OCP (38.5% vs. 16.3%; p = 0.006).

**Table 4:** Factors associated with cervical premalignant and malignant conditions (abnormal Pap test) (N = 280) Values are presented as n (%) or mean ± SD ^#^p-value was calculated using the Chi-squared test (for categorical variables) and Mann-Whitney U test (for age) *p < 0.05 is considered statistically significant STI: sexually transmitted infection; OCP: oral contraceptive pills; SD: standard deviation

Characteristics	Total	Normal/benign inflammation (n = 254)	Abnormal Pap test (n = 26)	p-value^#^
Age (years)	-	38.5 ± 9.1	39.8 ± 10.3	0.487
Residence				
Rural	163	145 (57.1)	18 (69.2)	0.232
Urban	117	109 (42.9)	8 (30.8)	
Literacy				
Literate	134	127 (50.0)	7 (26.9)	0.015*
Illiterate	146	127 (50.0)	19 (73.1)	
Socioeconomic status				
Class I/II/III	87	83 (32.7)	4 (15.4)	0.042*
Class IV/V	193	171 (67.3)	22 (84.6)	
Parity				
Nulliparous/primiparous	65	59 (23.2)	6 (23.1)	0.984
Multiparous	215	195 (76.8)	20 (76.9)	
Teenage pregnancy				
Yes	35	28 (11.0)	7 (26.9)	0.018*
No	245	226 (89.0)	19 (73.1)	
Multiple sexual partners				
Yes	10	6 (2.4)	4 (15.4)	0.003*
No	270	248 (97.6)	22 (84.6)	
History of STI				
Yes	25	17 (6.7)	8 (30.8)	<0.001*
No	255	237 (93.3)	18 (69.2)	
Long-term OCP use				
Yes	51	41 (16.1)	10 (38.5)	0.006*
No	229	213 (83.9)	16 (61.5)	

## Discussion

The mean age of participants was 38.6 ± 9.2 years, with the majority aged 31-40 years, consistent with previous studies [11-13]. This distribution reflects peak reproductive years when women experience cumulative exposure to risk factors, including human papillomavirus (HPV) infection, hormonal influences, and consequences of multiple pregnancies, underscoring the importance of targeted screening programs for women in their third and fourth decades.

A striking finding in our study was that more than half of the participants (58.2%) were from rural areas, where women face multiple barriers, including geographical distance from facilities, lack of transportation, financial constraints, and limited health literacy. This emphasizes the urgent need for mobile screening units, community-based education programs, and strengthened primary healthcare infrastructure in rural areas.

Over half of the participants were illiterate and belonged to lower socioeconomic classes, consistent with studies establishing strong associations between low socioeconomic status, illiteracy, and increased cervical cancer risk [14]. Lower socioeconomic status is linked to poor hygiene practices, limited healthcare access, delayed presentation, higher prevalence of concurrent infections, malnutrition, and reduced awareness about preventive measures. Illiteracy compounds these challenges by limiting women's ability to understand health information and navigate healthcare systems effectively.

The vast majority were multiparous. Multiparity increases cervical cancer risk through hormonal changes during pregnancy, cervical trauma during deliveries, pregnancy-associated immunosuppression, and prolonged HPV exposure. Muñoz et al. [15] demonstrated a dose-response relationship, with women having seven or more pregnancies showing a 3.8-fold increased risk compared to nulliparous women.

Teenage pregnancy, defined as first pregnancy before 18 years of age, was reported by 12.5% of participants in our study. Early age at first pregnancy is a well-established risk factor for cervical cancer, as it often serves as a marker for early initiation of sexual activity and prolonged exposure to HPV infection [16]. The cervix during adolescence undergoes active metaplastic transformation in the transformation zone, making it particularly vulnerable to HPV infection and subsequent neoplastic changes [16].

In our study, a history of multiple sexual partners was reported by 3.6% of participants, while 8.9% reported a past history of STIs. These figures likely represent underreporting due to social desirability bias and cultural stigma associated with discussing sexual behavior, particularly in conservative Indian society, where such topics are often considered taboo. Liu et al. [17], in their meta-analysis, found that women with multiple sexual partners had a significantly elevated risk of cervical intraepithelial neoplasia and cervical cancer.

Long-term OCP use (≥5 years) was reported by 18.2% of participants. The association between prolonged OCP use and cervical cancer has been documented in multiple studies, with meta-analyses showing a modest but consistent increase in risk [18]. The International Collaboration of Epidemiological Studies found relative risk of cervical cancer increased with OCP duration, though the risk diminished after discontinuation [19].

White vaginal discharge was the most common complaint (54.3%), consistent with previous studies [13]. High prevalence reflects the inflammatory nature of cervical lesions, concurrent infections, cervical ectopy, and increased mucus production. However, discharge is non-specific and can result from both benign and serious conditions. Abnormal vaginal bleeding was also highly prevalent in our study, with intermenstrual bleeding reported by 28.6%, post-coital bleeding by 15.4%, and post-menopausal bleeding by 12.5% of participants. These bleeding patterns are classic warning signs that should prompt a thorough investigation for cervical pathology. Post-coital bleeding, in particular, has high specificity for cervical lesions, as it results from contact trauma to friable cervical tissue. Post-menopausal bleeding requires especially careful evaluation, as it carries a higher likelihood of malignancy due to the atrophic changes in the genital tract that occur after menopause, making the tissue more susceptible to trauma and infection. Our findings emphasize the importance of comprehensive gynecological examination and appropriate screening for all women presenting with these symptoms.

Pap test examination revealed 62.5% had benign inflammatory changes, 28.2% normal cytology, and 9.3% abnormal results (ASCUS, LSIL, and HSIL). This prevalence of abnormal cytology is higher than what would be expected in a general screening population but is consistent with studies examining symptomatic women or those with a clinically unhealthy cervix [9]. The high proportion of inflammatory Pap smears in our study reflects the burden of cervicovaginal infections in the study population and highlights a significant limitation of cervical cytology in resource-limited settings. Inflammation can obscure cellular details and lead to false-negative results, potentially masking underlying dysplastic changes. This observation supports the argument for combining cytology with other screening modalities such as visual inspection with acetic acid (VIA) or HPV testing in symptomatic women to improve detection rates.

Our analysis revealed significant associations between sociodemographic/reproductive factors and premalignant lesions. The association between lower socioeconomic status and abnormal results (p = 0.042) reflects multiple barriers: financial constraints, lack of transportation, competing survival priorities, and limited household decision-making autonomy [14]. The association with illiteracy (p = 0.015) reflects the fundamental role of education in health-seeking behavior. Illiterate women have less awareness of screening options, difficulty understanding health information, challenges navigating healthcare, and greater reliance on traditional practices [20].

The significant associations observed with reproductive and sexual health factors, namely, teenage pregnancy, multiple sexual partners, and history of STIs, are all mechanistically linked to HPV exposure and persistence, the central etiologic factor in cervical carcinogenesis. These findings support the importance of comprehensive sexual health education, promotion of delayed sexual debut, encouragement of barrier contraceptive methods, effective STI prevention and treatment programs, and HPV vaccination for adolescents before sexual debut. The association with long-term OCP use (p = 0.006) adds to the growing body of evidence suggesting that prolonged hormonal contraceptive use may modestly increase cervical cancer risk, particularly in HPV-infected women [18]. This finding should inform contraceptive counseling, emphasizing the importance of regular cervical screening for long-term OCP users while acknowledging the important role of OCPs in preventing unintended pregnancies.

Our study has some limitations that should be acknowledged. The hospital-based design may have introduced selection bias, as women presenting to tertiary care facilities may differ systematically from the general population in terms of disease severity, health-seeking behavior, and socioeconomic characteristics. The cross-sectional design precludes assessment of temporal relationships and causal inferences. Self-reported data on sensitive topics such as sexual history and STI history may be subject to recall bias and social desirability bias, potentially leading to underreporting. Additionally, the study did not include HPV testing, which would have provided valuable information about the prevalence and types of HPV infections in this population. Consequently, the findings should be interpreted with caution, and their generalizability to the broader population may be limited.

## Conclusions

This study provides comprehensive data on women presenting with an unhealthy cervix at tertiary care facilities in Central and Eastern India, demonstrating a high burden of cervical pathology, particularly among rural, illiterate, and lower socioeconomic status women. Multiple modifiable risk factors were identified as significantly associated with premalignant lesions. These findings emphasize the urgent need for comprehensive cervical cancer prevention strategies, including population-based screening programs, targeted high-risk interventions, health education campaigns, strengthened rural healthcare infrastructure, and integration of screening with reproductive health services. Future research should evaluate the effectiveness and cost-effectiveness of different screening strategies in diverse Indian populations, assess barriers to screening uptake and follow-up, and develop culturally appropriate interventions to improve awareness and coverage. Only through such multi-pronged approaches can we reduce the substantial burden of cervical cancer in India and achieve the global goal of cervical cancer elimination.

## References

[1] Mehrotra R, Yadav K (2022). Cervical cancer: formulation and implementation of Govt of India guidelines for screening and management. Indian J Gynecol Oncol.

[2] (2024). World Health Organization. Factsheet: cervical cancer. https://www.who.int/news-room/fact-sheets/detail/cervical-cancer.

[3] Shamsunder S, Verma S (2024). Cervical cancer prevention efforts in India: a reality check. Journal of Colposcopy and Lower Genital Tract Pathology.

[4] Dasgupta A, Sinha RN, Paul B, Bandyopadhyay L, Banerjee R, Suman S (2019). A study on unhealthy cervix and its risk factors among currently married women of reproductive age group attending an urban health centre of Kolkata. Int J Community Med Public Health.

[5] Bobdey S, Sathwara J, Jain A, Balasubramaniam G (2016). Burden of cervical cancer and role of screening in India. Indian J Med Paediatr Oncol.

[6] Srivastava AN, Misra JS, Srivastava S, Das BC, Gupta S (2018). Cervical cancer screening in rural India: status & current concepts. Indian J Med Res.

[7] Pimple SA, Mishra GA (2019). Global strategies for cervical cancer prevention and screening. Minerva Ginecol.

[8] Gupta R, Gupta S, Mehrotra R, Sodhani P (2017). Cervical cancer screening in resource-constrained countries: current status and future directions. Asian Pac J Cancer Prev.

[9] Najib FS, Hashemi M, Shiravani Z, Poordast T, Sharifi S, Askary E (2020). Diagnostic accuracy of cervical Pap smear and colposcopy in detecting premalignant and malignant lesions of cervix. Indian J Surg Oncol.

[10] Nayar R, Wilbur DC (2017). The Bethesda system for reporting cervical cytology: a historical perspective. Acta Cytol.

[11] Venkatesh M, Gopalan U (2020). A comparative study of Pap smear and colposcopy guided biopsy in the evaluation of unhealthy cervix. Int J Reprod Contracept Obstet Gynecol.

[12] Subramanyam BR, Garlapati H (2020). Comparative study of pap smear and colposcopy guided biopsy in a district medical college. Int J Community Med Public Health.

[13] Kohale MG, Dhobale AV, Hatgoankar K, Bahadure S, Salgar AH, Bandre GR (2024). Comparison of colposcopy and histopathology in abnormal cervix. Cureus.

[14] Sultana S, Khatun S (2025). Cervical cancer in Asian countries: epidemiology, risk factors, and challenges. Int J Reprod Contracept Obstet Gynecol.

[15] Muñoz N, Franceschi S, Bosetti C (2002). Role of parity and human papillomavirus in cervical cancer: the IARC multicentric case-control study. Lancet.

[16] Chelimo C, Wouldes TA, Cameron LD, Elwood JM (2013). Risk factors for and prevention of human papillomaviruses (HPV), genital warts and cervical cancer. J Infect.

[17] Liu ZC, Liu WD, Liu YH, Ye XH, Chen SD (2015). Multiple sexual partners as a potential independent risk factor for cervical cancer: a meta-analysis of epidemiological studies. Asian Pac J Cancer Prev.

[18] Asthana S, Busa V, Labani S (2020). Oral contraceptives use and risk of cervical cancer-a systematic review & meta-analysis. Eur J Obstet Gynecol Reprod Biol.

[19] (2007). Cervical cancer and hormonal contraceptives: collaborative reanalysis of individual data for 16 573 women with cervical cancer and 35 509 women without cervical cancer from 24 epidemiological studies. Lancet.

[20] Taneja N, Chawla B, Awasthi AA, Shrivastav KD, Jaggi VK, Janardhanan R (2021). Knowledge, attitude, and practice on cervical cancer and screening among women in India: a review. Cancer Control.

